# Global genome analysis of the shikimic acid pathway reveals greater gene loss in host-associated than in free-living bacteria

**DOI:** 10.1186/1471-2164-11-628

**Published:** 2010-11-11

**Authors:** Jurica Zucko, Walter C Dunlap, J Malcolm Shick, John Cullum, François Cercelet, Bijal Amin, Lena Hammen, Timothy Lau, Jamal Williams, Daslav Hranueli, Paul F Long

**Affiliations:** 1 Department of Genetics, University of Kaiserslautern, Postfach 3049, 67653 Kaiserslautern, Germany; 2 Section for Bioinformatics, Department of Biochemical Engineering, Faculty of Food Technology and Biotechnology, University of Zagreb, Pierottijeva 6, 10000 Zagreb, Croatia; 3 Centre for Marine Microbiology and Genetics, Australian Institute of Marine Science, PMB 3 Townsville MC, Townsville, Queensland, 4810 Australia; 4 School of Marine Sciences, University of Maine, 5751 Murray Hall, Orono, ME 04469-5751 USA; 5 The School of Pharmacy, University of London, 29/39 Brunswick Square, London WC1N 1AX, UK; 6 Department of Bioengineering, Polytech'Nice-Sophia, 930 Route des Colles, 145-06903, Sophia Antipolis, Cedex, France; 7 Institute of Pharmacy and Molecular Biotechnology, University of Heidelberg, Im Neuenheimer Feld 364, D-69120 Heidelberg, Germany

## Abstract

**Background:**

A central tenet in biochemistry for over 50 years has held that microorganisms, plants and, more recently, certain apicomplexan parasites synthesize essential aromatic compounds via elaboration of a complete shikimic acid pathway, whereas metazoans lacking this pathway require a dietary source of these compounds. The large number of sequenced bacterial and archaean genomes now available for comparative genomic analyses allows the fundamentals of this contention to be tested in prokaryotes. Using Hidden Markov Model profiles (HMM profiles) to identify all known enzymes of the pathway, we report the presence of genes encoding shikimate pathway enzymes in the hypothetical proteomes constructed from the genomes of 488 sequenced prokaryotes.

**Results:**

Amongst free-living prokaryotes most Bacteria possess, as expected, genes encoding a complete shikimic acid pathway, whereas of the culturable Archaea, only one was found to have a complete complement of recognisable enzymes in its predicted proteome. It may be that in the Archaea, the primary amino-acid sequences of enzymes of the pathway are highly divergent and so are not detected by HMM profiles. Alternatively, structurally unrelated (non-orthologous) proteins might be performing the same biochemical functions as those encoding recognized genes of the shikimate pathway. Most surprisingly, 30% of host-associated (mutualistic, commensal and pathogenic) bacteria likewise do not possess a complete shikimic acid pathway. Many of these microbes show some degree of genome reduction, suggesting that these host-associated bacteria might sequester essential aromatic compounds from a parasitised host, as a 'shared metabolic adaptation' in mutualistic symbiosis, or obtain them from other consorts having the complete biosynthetic pathway. The HMM results gave 84% agreement when compared against data in the highly curated BioCyc reference database of genomes and metabolic pathways.

**Conclusions:**

These results challenge the conventional belief that the shikimic acid pathway is universal and essential in prokaryotes. The possibilities that non-orthologous enzymes catalyse reactions in this pathway (especially in the Archaea), or that there exist specific uptake mechanisms for the acquisition of shikimate intermediates or essential pathway products, warrant further examination to better understand the precise metabolic attributes of host-beneficial and pathogenic bacteria.

## Background

**Figure 1:**
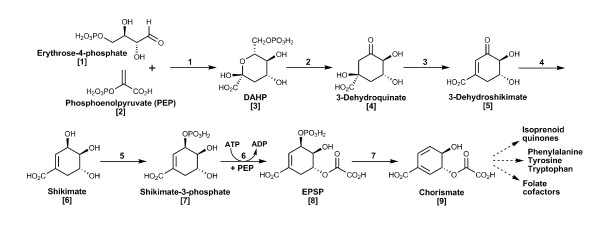
**Schematic representation of the shikimic acid pathway**. The first step is an aldol condensation between phosphoenol pyruvate [1] and erythrose-4-phosphate [2] catalyzed by 3-deoxy-D-arabinoheptulosonate-7-phosphate synthase (DAHP, or aroG/F/H according to *E. coli *nomenclature, EC 4.1.2.15) to synthesize 3-deoxy-D-arabino-heptulosonate-7-phosphate [3]. The second step is carried out by 3-dehydroquinate synthase (DHQ synthase, aroB, EC 4.6.1.3), affording 3-dehydroquinate [4]. The third step synthesizes 3-dehydroshikimate [5] catalyzed by 3-dehydroquinate dehydratase (DH-quinase, aroD, EC 4.2.1.10). Step 4 is catalyzed by shikimate dehydrogenase (Shikimate_DH, aroE, EC 1.1.1.25) to synthesize shikimate [6]. Steps 5 and 6 are catalyzed by shikimate kinase (SKI, aroK, EC 2.7.1.71) and 5-enolpyruvylshikimate-3-phosphate synthase (EPSP, aroA, EC 2.5.1.19) to give shikimate-3-phosphate [7] and 5-enolpyruvylshikimate-3-phosphate [8], respectively. The last reaction, catalyzed by chorismate synthase (aroC, EC 4.6.1.4) affords the final product of the pathway, chorismate [9].

Chorismic acid is the direct precursor of many aromatic compounds, including aromatic amino acids, folate, ubiquinones and other isoprenoid quinones [1]. The biosynthesis of chorismic acid occurs via the shikimic acid pathway in the seven enzyme-mediated steps shown in Figure 1. Considered restricted to bacteria, fungi, yeasts, algae, plants and certain apicomplexan parasites, the lack of a shikimic acid pathway in metazoans, including humans, is evinced by their dietary requirements for aromatic compounds. The universality of this traditional view has been challenged previously by our finding of genes encoding enzymes for the shikimic acid pathway in the genome of a basal metazoan, the starlet sea anemone *Nematostella vectensis*, a cnidarian [2]. Bioinformatic analyses established that horizontal transfer of ancestral genes of the shikimic acid pathway into the *N. vectensis *genome occurred from both bacteria and algae (a dinoflagellate). Molecular evidence suggesting also the presence of an unsuspected bacterial symbiont in this sea anemone gives a complementary view for the biogenesis of shikimate-related metabolites as a "shared metabolic adaptation" between the symbiotic partners [2]. Similarly, analysis of the genomes of aphids reveals genes acquired from endosymbiotic bacteria by lateral transfer, genes that after subsequent deletion by the bacterial symbiont underlie the obligate nature of the metabolic relationship in *Buchnera *spp. [3].

In a comparative genomic study of four thermophilic microorganisms (*Aquiflex aeolicus*, *Archaeoglobus fulgidus*, *Methanobacterium thermoautotrophicum *and *Methanococcus jannaschii*), genes encoding DAHP synthase and DHQ synthase (the first two steps in the pathway) appear missing from these archaeans [4]. For genes to be missing from an essential biosynthetic pathway might be accounted for by the presence of genes having low sequence similarity to the known genes, or substitution by alternative enzymes having an analogous function. Another possibility is that steps of a biosynthetic pathway may be bypassed if substrates and end-products are readily obtained from the surrounding environment [5]. Evolutionary pressures would then lead to loss of genes encoding discrete elements of the pathway, which is an underlying cause of extreme genome reduction and instability as observed in the small genomes of some intracellular pathogens and symbionts, *e.g*. *Mycobacterium leprae*, *Rickettsia, Bartonella *and *Buchnera *[6-8]. The recent surge in microbial genome sequencing has produced a wealth of genetic data available for comparative genomic analyses to make possible the identification of diverse essential enzymes in critical metabolic pathways within the Archaea and Bacteria. Here we interrogate the hypothetical proteomes of prokaryotes, constructed from their published genomes, to profile the universality of the shikimic acid pathway with a view to understanding a key metabolic process of free-living and host-associated bacteria.

## Results

Hypothetical proteomes translated from the completed genomes of 488 prokaryotes listed in the NCBI database were interrogated using HMM profiles generated to describe enzymes of the pathway [Table 1]. There is a high degree of amino acid sequence diversity amongst the 3-dehydroquinate synthase and shikimate kinase enzymes. For this reason, two separate HMM models were generated for each of these enzymes and the best bit-scores, e-values and protein coverage were utilized for analysing the predicted microbial proteomes. A cut-off score of >50 with a stringent e-value of <10^-8 ^were required, together with protein coverage of >90%, for inclusion of an enzyme in the hypothetical proteome. It was expected that as an essential anabolic sequence, the shikimic acid pathway would be under strong selective constraints and that most prokaryotes would have a complete and recognizably conserved pathway. Surprisingly, this was not the case: although two thirds (69%) of the predicted prokaryotic proteomes contained a complete shikimic acid pathway, nearly one-third (31%) did not [Table 2].

**Table 1:** Templates used to interrogate shikimic acid pathway structure in the sequenced genomes of prokaryotes.

Shikimate Pathway Step	Product	Enzymes and Enzyme Isoforms	Source and Genetic/Protein Templates
1	3-Deoxy-D-arabino-heptulosinate -7- phosphate (DAHP)	DAHP synthase EC 4.1.2.15 (aroF), (aroG), (aroH)	In *E. coli *there are three DAHP synthetase isoforms, each specifically inhibited by one of the three aromatic amino acids.
	
		KDPGal aldolase EC 4.1.2.21	See: http://www.brenda-enzymes.org/php/result_flat.php4?ecno = 4.1.2.21 and [12]

2	3-Dehydroquinate (DHQ)	DHQ synthase EC 4.2.3.4 (aroB)	DHQ synthase exists as type 1 and 2 enzymes (previously EC 4.6.1.3)

3	3-dehydroshikimate (DHS)	Shikimate dehydrase EC 4.2.1.10 (aroD)	shikimate dehydrase and shikimate dehydrogenase are often a bifunctional enzyme

4	Shikimic acid (shikimate)	Shikimate dehydrogenase EC 1.1.1.25 (aroE)	*E. coli *has the putative enzyme YdiB paralog [25]

5	Shikimate-3-phosphate	Shikimate kinase II EC 2.7.1.71 (aroL)	monofunctional shikimate kinase
	
		Archaeal GHMP shikimate kinase	See: http://www.wikiproteins.org/index.php/Concept:62865969 and [11]

6	5-Enolpyruvyl-shikimate-3-phosphate (EPSP)	EPSP Synthase EC 2.5.1.19 (aroA)	The *AroA *gene, coding for the *E. coli *EPSP synthase, was first isolated from a lambda transducing phage (*lambda-serC*) found to contain a portion of the *E. coli *chromosome

2-6	5-Enolpyruvyl-shikimate-3-phosphate (EPSP)	Shikimate kinase I EC 4.2.3.4 EC 4.2.1.10 EC 1.1.1.25 EC 2.7.1.71 EC 2.5.1.19 (aroM)	Pentafunctional gene consisting of *aroB*, *aroD*, *aroE*, *aroL *and *aroA*

7	Chorismic acid (chorismate)	Chorismate synthesis EC 4.2.3.5 (aroC)	previously EC 4.6.1.4 Chorismate synthase from various sources shows a high degree of sequence conservation.

**Table 2:** The presence or absence of a complete shikimic acid pathway deduced from enzymes detected by HMM analysis in the predicted proteomes of 488 prokaryotes.

	Total	Complete pathway	Incomplete pathway	% incomplete
Total	488	336	152	31
Bacteria	442	335	107	24
Free living	147	131	16	11
Host-associated	295	204	91	31
Archaea	46	1	45	98

As expected, nearly all of the 147 Bacteria that have never been reported previously to be associated with a host (termed 'free-living') contain a complete complement of enzymes forming this pathway.

**Figure 2:**
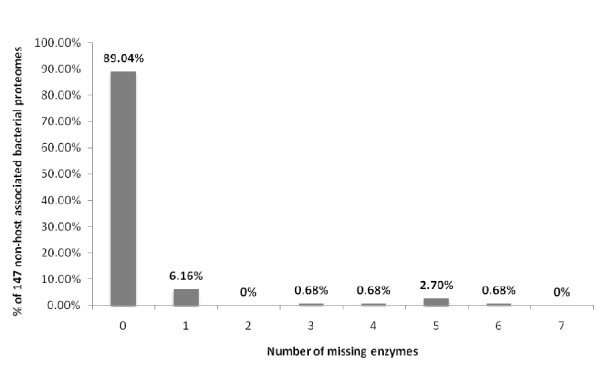
**HMM analysis of the shikimic acid pathway in the predicted proteomes of 147 'free-living' Bacteria shows that most have the complete pathway and only a few are missing one or more enzymes in the pathway**.

**Figure 3:**
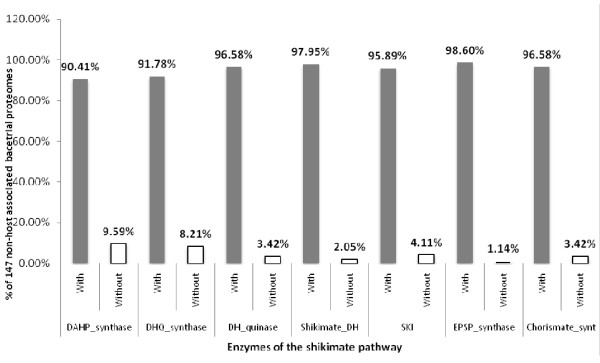
**HMM analysis showing the distribution of enzymes forming the shikimic acid pathway in the predicted proteomes of 147 'free-living' Bacteria; there is no pattern to which enzymes are missing**.

**Figure 4:**
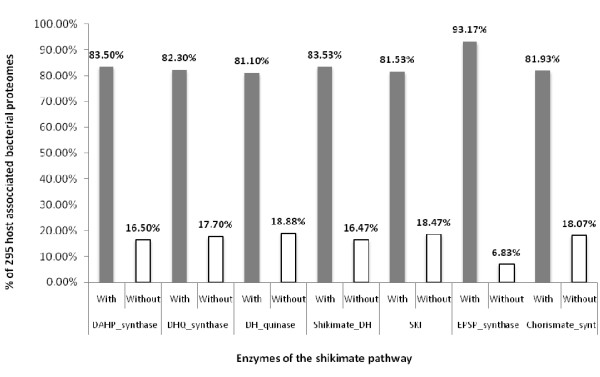
**HMM analysis showing the distribution of enzymes forming the shikimic acid pathway in the predicted proteomes of 250 host-associated Bacteria that can be cultured in the laboratory *ex hospite *shows that there is a nearly equal loss of enzymes across the pathway**.

**Figure 5:**
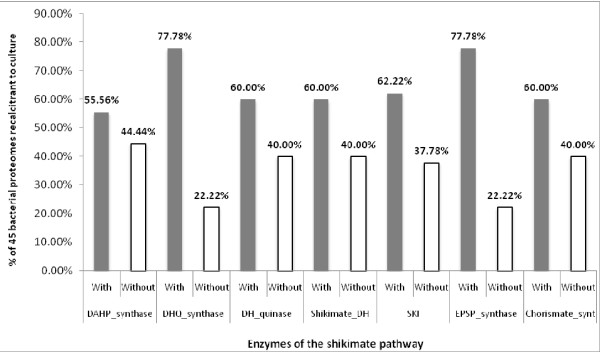
**HMM analysis of the distribution of enzymes forming the shikimic acid pathway in the predicted proteomes of 46 host-associated Bacteria that cannot be cultured *ex hospite *shows that there is a nearly equal loss of enzymes across the pathway**.

The proteomes were then divided according to the two prokaryotic domains, the Bacteria and the Archaea. The proteomes of Bacteria studied could be further divided into free-living and host-associated phenotypes. Of the free-living bacteria [Additional File 1], most as expected contained a complete pathway [Table 2], and those having an incomplete pathway were missing just one or two enzymes [Figure 2], although there was no pattern to which enzymes were absent [Figure 3]. Of the host-associated bacteria, which included pathogenic, commensal and mutualistic relationships [Additional File 2], surprisingly more than one-quarter (91/295 proteomes) of these bacteria had an incomplete pathway, missing one or more enzymes [Table 3]. Of these bacteria, most appear refractory to culture [Table 3]. As with the other prokaryotic proteomes studied, there was no overall pattern to which enzymes of the pathway were absent [Figures 4 and 5].

**Table 3:** The presence or absence of a complete shikimic acid pathway deduced from enzymes detected by HMM analysis in the predicted proteomes of 488 Bacteria.

	Total	Complete pathway	Incomplete pathway	% incomplete
Culturable	250	181	69	28
Non-culturable	45	23	22	49
Total	295	204	91	31

All bacteria are reported in the literature as host-associated (pathogenic, commensal or mutualistic). Species that are reported to yield to laboratory culture *ex hospite *were classed as culturable.

**Figure 6:**
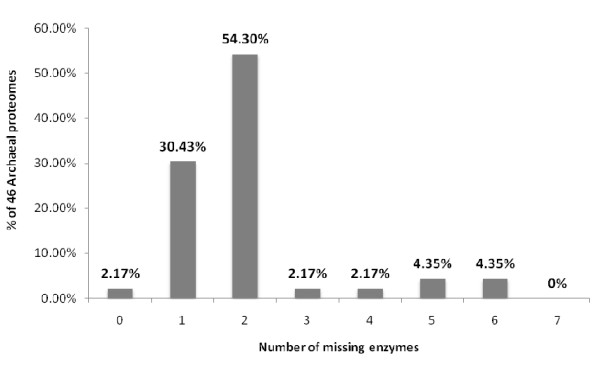
**HMM analysis of the shikimic acid pathway in the predicted proteomes of 46 Archaea shows that nearly all of these prokaryotes are missing only one or two enzymes in the pathway**.

**Figure 7:**
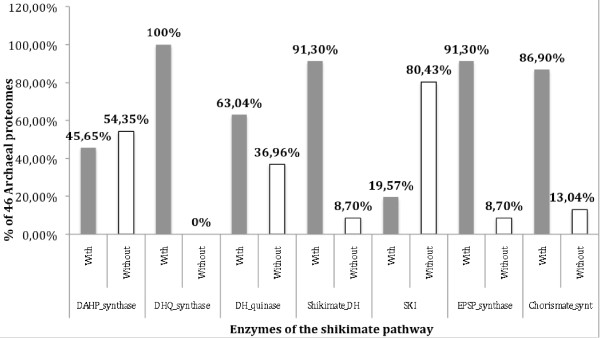
**HMM analysis showing the distribution of enzymes forming the shikimic acid pathway in the predicted proteomes of 46 Archaea; nearly all of these prokaryotes are missing either the first enzyme in the pathway, DAHP synthase, or the fifth enzyme, shikimate kinase**.

Of the Archaea sequencing projects to date, all of the selected microorganisms are free-living and have not been described as usually associated with a known host. Table 2 [and Additional File 3] shows that all except one (45/46) of the archaeans have an incomplete pathway, missing generally only one or two enzymes [Figure 6], predominantly either DAHP synthase or shikimate kinase [Figure 7]. Previous genomic surveys of the distribution of genes encoding the shikimic acid pathway in a limited number of archaeans have shown by sequence comparison methods that these two enzymes are frequently missing [4], in the case of shikimate kinase perhaps as a result of a non-orthologous displacement by an enzyme distantly related to homoserine kinases (EC 2.7.1.39) of the GHMP-kinase superfamily [9]. To test this hypothesis, a HMM profile for the GHMP kinases was developed and tested against the proteomes of Achaea, as well as free-living and host-associated Bacteria. The proteomes of all 488 prokaryotes were found to contain a serine kinase enzyme, however, it is likely that our model for GHMP kinases was not specific for the shikimate kinase previously reported [9] and instead identified all serine kinase enzymes encoded in the archaean genomes. It has also been shown in *Escherichia coli *that 2-keto-3-deoxy-6-phosphogalactonate (KDPGal) aldolase provides an alternative reaction to that catalyzed by DAHP synthase (the first step in the pathway) for the synthesis of 3-deoxy-D-arabino-heptulosonate-7-phosphate from phosphoenol pyruvate and erythrose-4-phosphate [10]. Analysis of the 14 *E. coli *proteomes available showed that all 14 contained DAHP synthase while 13/14 contained KDPGal aldolase. However, the HMM profile constructed to recognise KDPGal aldolase could only demonstrate the presence of this enzyme in 14 of the 108 prokaryotic proteomes from which DAHP synthase was missing [Additional Files 4, 5, 6].

Results were also checked against the BioCyc curated reference database of bacterial genomes and metabolic pathways [11]. Overall, the results were in agreement in 84% of cases (Additional Files 7, 8, 9). Of the 85 non-host associated bacteria represented in the database, 75 were predicted to contain a complete complement of shikimic acid pathway enzymes when compared to the HMM models. An additional 4 complete pathways were detected that were missed by the rapid, yet automated HMM search. The HMM search did, however, predict 4 complete pathways that were missing in the BioCyc database. Similarly, of the 204 host-associated bacteria that were evaluated using the BioCyc database, 168 were found to contain a complete shikimic acid pathway using both BioCyc and HMM searches, with 23 additional complete pathways detected using BioCyc and 12 additional complete pathways detected by the HMM search (Additional File 8). For the Archaea, 33 proteomes could be evaluated, of which 27 were common to both searches with 5 additional complete pathways found in the BioCyc database and 1 using the HMM search (Additional File 9). In searching the human genome for genes encoding enzymes of the shikimic acid pathway, only one previously annotated gene was found when interrogated using HMM profiles. This alignment gave a score of 149 with an e-value of 10^-44^, but corresponded only to the ATP-binding domain of shikimate kinase encoded on chromosome 10 (accession number NT_008705.15).

## Discussion

The shikimic acid pathway is well recognized in classical biochemistry to be essential for the synthesis of aromatic compounds in prokaryotes, fungi, certain apicomplexans and plants [1]. The lack of the shikimic acid pathway in metazoans, most notably humans as evinced by our dietary requirement for shikimate-derived aromatic compounds, has stimulated much study of this pathway as a possible target for antimicrobial chemotherapy [12]. The emergence of microbial pathogens resistant to many drugs in our current pharmacopeia has prompted widespread efforts to identify suitable novel targets for the design of antimicrobial drugs that lack untoward side effects and, since the pathway is lacking in humans, forms a rational basis for drug selectivity in lead target identification [13,14]. Accordingly, the structure and evolution of this pathway in eukaryotes has been comprehensively investigated [15], although conservation of the pathway in prokaryotes has not been subjected to widespread comparative genomic analysis. A pan-genomic bioinformatics evaluation of the conservation of enzymes forming the shikimic acid pathway in prokaryotes was therefore undertaken using automated HMM searching, leading to the unexpected result that nearly one-third of all prokaryotes examined lack a complete, recognizable pathway [Table 2]. Our results were comparable to data in the comprehensively curated BioCyc database (Additional Files 7, 8, 9). Data, however, had to be extracted from this database manually which proved labour intensive and time consuming, nevertheless useful for method validation.

Expression of the shikimic acid pathway is regulated via feedback inhibition by pathway intermediates and downstream products [16]. Although a variety of functional, biophysical, and fitness-related variables influence the evolutionary rates of proteins [17], the level of gene expression is one of the major determinants [18]. If a protein is highly expressed, its overall indispensability to the organism is greater than if it were expressed only at low levels, so that the functionally active amino acid residues of the protein would be under strong purifying selection [19]. Such selection on a large number of these protein residues leads to an overall reduced evolutionary rate and overall conservation of the metabolic pathway, since mutations in essential proteins are apt to be deleterious [20]. Regulation of the shikimic acid pathway can, therefore, be coupled to the exogenous availability of products of its component enzymes, giving a positive selective force leading to the loss of pathway genes. This follows from the surprising result that large numbers of host-associated bacteria lack a complete shikimic acid pathway.

Many of these bacteria are associated with the human microbiome, but no enzymes of the shikimic acid pathway could be detected using the HMM profiles on the translated human genome. This supports current dogma that the human host does not synthesize shikimate-derived aromatic compounds *de novo*, and leads to the strong inference that human-associated heterotrophic bacteria having genomes that encode an incomplete shikimic acid pathway may have evolved highly efficient means of extracting essential shikimate-related metabolites from their microbial environment. In symbiosis this could be from trophically derived metabolites assimilated by the host or from metabolites produced by other bacterial consorts having a complete and functional shikimic acid pathway. Uptake mechanisms for intermediates in the shikimic acid pathway and for some of the products of chorismate-utilizing enzymes are known in bacteria. For example, the shikimate permease ShiA [21], various aromatic amino acid permeases [22], and transporters for vitamins [23] and folic acid [24] are known, but the full phylogenetic distribution of these uptake systems and their relevance in complementing shikimic acid pathway-depleted prokaryotes are yet to be determined. Sequestering of other shikimate-derived metabolites, for example, ubiquinones, menaquinones, iron chelating siderophores and vitamins remains unknown. Substituting these essential metabolites into synthetic growth media might be one approach to successfully culturing those symbionts so far refractory to laboratory culture *ex hospite*.

On examination of the 91 host-associated bacteria lacking a complete pathway in detail [Additional File 1], most (67/91) had lost five or more of the genes encoding enzymes of the shikimic acid pathway, whereas nearly all of the rest (20/91) have only lost a single enzyme. In the entire set of host-associated bacteria, genes encoding the seven different enzymes are lost rather uniformly [Figure 4], with the first enzyme of the pathway accounting for only 67/440 of lost genes. However, in the 20 host-associated bacteria that have only lost one gene, the majority (16/20) have lost the gene encoding the first enzyme of the pathway. This pattern would be expected if selection were occurring under conditions in which the pathway was induced, because a later block might result in the accumulation of redundant intermediates of the pathway, which would likely be deleterious for the bacterium. A possible scenario is that functional loss of the shikimic acid pathway could be an early step toward sustaining a host-associated life style in which bacteria are prevented from outgrowing their hosts in times of nutritional stress.

The phylogenetically widespread and differential lack of orthologous genes encoding shikimic acid pathway enzymes in free-living Archaea [Figures 6, 7] seems unlikely to be circumvented by evolving specific uptake mechanisms for essential aromatic compounds since metabolites derived from the shikimic acid pathway are known to be limiting in natural environments. Indeed, the presence of these compounds secreted by bacteria can act as predatory chemoattractants for soil amoebae [24]. Given the variability in which particular enzymes are missing from such a wide sample of the Archaea and host-associated Bacteria, cultivable or not, there is no easy genetic explanation for this loss, since the genes encoding individual enzymes of the shikimate pathway are not clustered in these prokaryotes [Additional File 10]. In bacteria there is evidence for the intriguing possibility that in pathway equilibrium, lost intermediates from "missing" enzymic reactions could be supplied by reverse biosynthesis, as was demonstrated in *E. coli *for quinic and dehydroquinic acids derived from shikimic acid uptake [25].

Reductive evolution is the process whereby host-associated consorts decrease their genome size by abandoning genes that are needed by free-living microorganisms but that are dispensable when living in association if essential gene products are readily available from the host or from other symbiotic partners. A domino effect would follow: the more enzymes that are lost, the less likely are bacteria able to survive without the provision of shikimate pathway intermediates or end-products, driving survival toward obligate symbiotic associations and loss of the metabolic independence needed for culture *ex hospite*. This scenario is especially true for the accelerated evolution of endosymbiotic lineages as expected by the combined effects of the accumulation of irreversible mutations (Muller's ratchet) and mutational bias [26].

The most obvious explanation for "missing" enzymes is the existence of functionally equivalent proteins that lack homology to the HMM models used in this study. Examples of non-orthologous gene replacements encoding enzymes that catalyze the same reaction indeed are known for the shikimic acid pathway and were tested in this study. These include the first step catalyzed by DAHP synthase [13], and the fifth step, catalyzed by shikimate kinase [12]. However, in our study there was no evidence based on HMM profiling using the non-orthologous proteins as models to indicate that such non-orthologous enzymes replaced those missing in the prokaryotes studied, which strongly suggests that other enzymes that have yet to be identified may fill these gaps [Additional File 4, 5, 6]. This would suggest that, at least in the Archaea, these prokaryotes can synthesize aromatic compounds by a novel biochemical pathway that is yet to be discovered. Indeed, examining the nutritional requirements of the Archaea, as evinced by a survey of the growth media recommended by the DSMZ culture collection http://www.dsmz.de/, reveals that most of the favoured media are minimal, lacking exogenous aromatic amino acids.

## Conclusions

This comparative bioinformatics analysis of genes of the shikimic acid pathway in prokaryotes provides essential details that should help guide the choice of key organisms for future studies designed to reveal new metabolic processes in shikimic acid biosynthesis or to validate the loss of key biosynthetic genes. Such studies will undoubtedly provide new insight into the evolutionary history of the shikimic acid pathway that is particularly important in understanding how pathogenic bacteria synthesize or acquire shikimate-derived products and thus to identify new targets for antibiotic treatment.

## Methods

This study analysed the completed genome sequences of organisms on the NCBI Microbial Genome Projects page http://www.ncbi.nlm.nih.gov/genomes/lproks.cgi listed on June 3, 2009. The genomes were grouped using the 'all bacteria' and 'all archaea' selection tool and then filtered using the 'organism info' page to select for symbiotic prokaryotes using the key words 'disease', 'pathogenic in' and 'habitat - host associated'. Free-living prokaryotes were taken as all other entries with the key words 'habitat - multiple, aquatic, specialized or terrestrial'. The predicted proteomes from these genomes for each of the prokaryotes were downloaded from the FTP server at the NCBI ftp://ftp.ncbi.nih.gov/genomes/Bacteria/. The DNA sequences of all 23 haploid chromosomes and mitochondrial DNA of the human genome http://www.ncbi.nlm.nih.gov/sites/entrez?Db=genomeprj&cmd=ShowDetailView&TermToSearch=9558 were translated into all six reading frames using Transeq http://www.ebi.ac.uk/emboss/transeq. For profile analyses, HMMER version 2.3.2 http://hmmer.janelia.org and release 20 of the Pfam database http://www.sanger.ac.uk/Software/Pfam were used. HMM profiles for each of the enzymes of the shikimic acid pathway, KDPGal aldolase, GHMP kinase [see also Table 1], the hypothetical proteomes of the 486 prokaryotes analyzed, and a script written in Bioperl to manipulate output from HMMER analyses are available as flat files at the server http://bioserv2.pbf.hr/bmc/. Results were compared against existing metabolic pathway annotations available for 322 prokaryote proteomes in the BioCyc database http://biocyc.org/.

## Authors' contributions

WCD, JMS, DH, JC, PFL designed the research, JZ, FC, BA, LH, TL, JW, PFL performed the research, all of the authors analyzed the data and wrote the paper. All authors read and approved the final manuscript.

## Supplementary Material

Additional file 1 **Distribution of genes encoding enzymes of the shikimic acid pathway in non-host associated (free-living) Bacteria**. Click here for file

Additional file 2 **Distribution of genes encoding enzymes of the shikimic acid pathway in host-associated Bacteria**. Click here for file

Additional file 3 **Distribution of genes encoding enzymes of the shikimic acid pathway in Archaea**. Click here for file

Additional file 4 **Distribution of the gene encoding KDPGal aldolase in non-host-associated (free-living) Bacteria**. Click here for file

Additional file 5 **Distribution of the gene encoding KDPGal aldolase in host- associated Bacteria**. Click here for file

Additional file 6 **Distribution of the gene encoding KDPGal aldolase in Archaea**. Click here for file

Additional file 7 **Comparison of the distribution of the shikimic acid pathway in 85 non-host associated bacteria using HMM and BioCyc database searching**. Click here for file

Additional file 8 **Comparison of the distribution of the shikimic acid pathway in 204 host-associated bacteria using HMM and BioCyc database searching**. Click here for file

Additional file 9 **Comparison of the distribution of the shikimic acid pathway in 33 Archaea using HMM and BioCyc database searching**. Click here for file

Additional file 10 **Genetic architecture of the shikimic acid pathway across a range of taxonomically different prokaryote genomes shows that the genes encoding the pathway are not clustered**. Click here for file
